# Expression profiling of circulating non-red blood cells in embryonic blood

**DOI:** 10.1186/1471-213X-8-21

**Published:** 2008-02-27

**Authors:** Brendan AS McIntyre, Cantas Alev, Hiroshi Tarui, Lars M Jakt, Guojun Sheng

**Affiliations:** 1 Laboratory for Early Embryogenesis, RIKEN Center for Developmental Biology, Kobe, Hyogo 650-0047, Japan; 2 Stem Cell Translational Research, Institute for Biomedical Research and Innovation, Kobe, Hyogo 650-0047, Japan; 3 Genome Resource and Analysis Unit, RIKEN Center for Developmental Biology, Kobe, Hyogo 650-0047, Japan; 4 Laboratory for Stem Cell Biology, RIKEN Center for Developmental Biology, Kobe, Hyogo 650-0047, Japan

## Abstract

**Background:**

In addition to erythrocytes, embryonic blood contains other differentiated cell lineages and potential progenitor or stem cells homed to changing niches as the embryo develops. Using chicken as a model system, we have isolated an enriched pool of circulating non red blood cells (nRBCs) from E4 and E6 embryos; a transition period when definitive hematopoietic lineages are being specified in the peri-aortic region.

**Results:**

Transcriptome analysis of both nRBC and RBC enriched populations was performed using chicken Affymetrix gene expression arrays. Comparison of transcript profiles of these two populations, with verification by RT-PCR, reveals in nRBCs an expression signature indicative of hematopoietic stem cells (HSCs) and progenitor cells of myeloid and lymphoid lineages, as well as a number of previously undescribed genes possibly involved in progenitor and stem cell maintenance.

**Conclusion:**

This data indicates that early circulating embryonic blood contains a full array of hematopoietic progenitors and stem cells. Future studies on their heterogeneity and differentiation potentials may provide a useful alternative to ES cells and perinatal blood.

## Background

The isolation and gene expression profiling of embryonic circulating nRBCs would be of great interest to developmental biologists and clinicians alike [1], yet due to limited sample size available from traditionally used model organisms, harvesting a sufficiently large pool of embryonic nRBCs for transcriptome-wide analysis has been difficult. Alternative approaches using perinatal blood have already yielded significant insights [2]. The chick embryo is both large in size and contains a circulatory network of a complexity equal to that of mammals. Herein, we describe the isolation and gene expression profiling of circulating cells during the transition phase of hematopoiesis from primitive or yolk sac associated, to definitive hematopoiesis, at embryonic days 4 and 6. It is during this time that hematopoiesis occurs transiently in the peri-aortic region in the chick embryo (referred to in mammals as the aorta-gonad-mesonephros or AGM), before transitioning to the bone marrow [3,4].

## Results and Discussion

**Figure 1:**
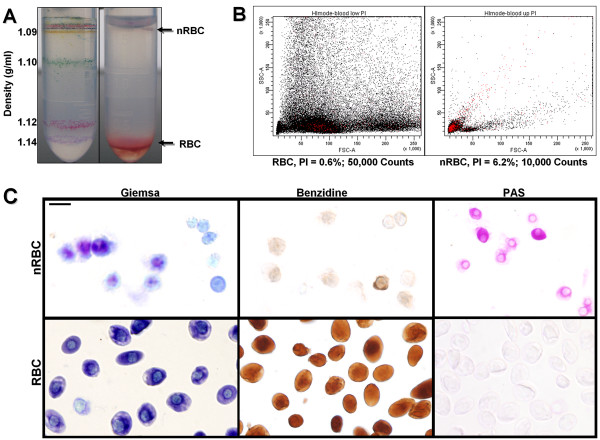
Characterization of RBC and nRBC cellular fractions. (A) Appearance of cell populations following density gradient centrifugation, along with control density marker beads. (B) FACSAria (BD Biosciences) profile of RBC and nRBCs after propidium iodide labeling of dead cells. The number of small (FSC), agranular (SSC) cells is greater in the nRBC fraction; >90% of cells from both populations are viable based on propidium iodide exclusion and Trypan blue exclusion (not shown). (C) nRBCs have a large nuclear volume, and smaller size (Giemsa), are benzidine negative and PAS positive. Bar = 20 μm.

Chick blood was isolated from embryos at E4 and E6, using micro-capillaries inserted directly into the heart. Density gradient centrifugation was then employed to isolate the heavier RBCs from a lighter nRBC population from total embryonic blood. Cells within the two populations were analyzed directly by FACS, and by the classical hematological stains Giemsa, benzidine, and Periodic-acid Schiff (PAS). Using these techniques, we were able to confirm that two distinct, viable populations; one highly enriched in RBCs, and another population highly depleted of RBCs (nRBCs) had been isolated (Fig. 1).

**Figure 2:**
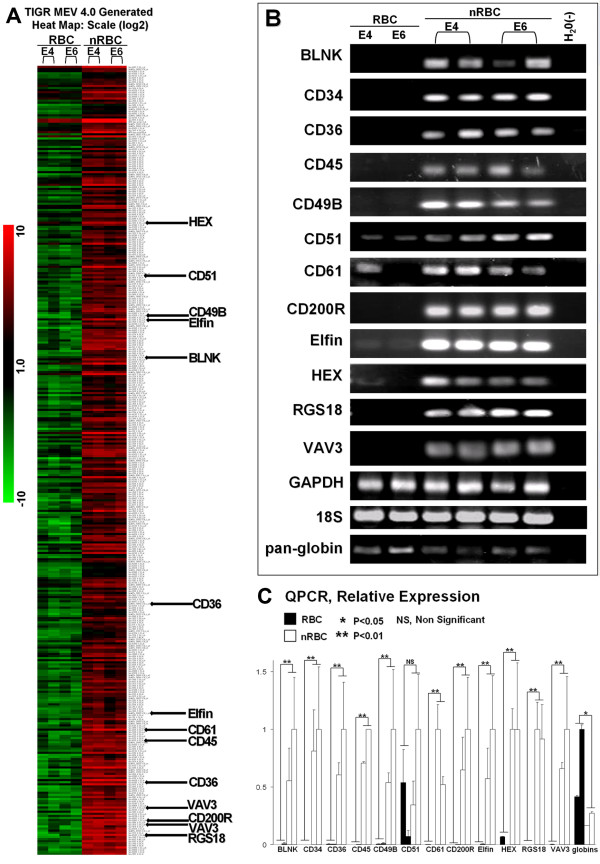
Array analysis and RT-PCR verification. (A) Heat map generated by TM4 SAM analysis with genes verified by PCR highlighted. (B) Semi-quantitative PCR analysis of candidate genes from array data and control GAPDH and 18S, and embryonic hemoglobin transcripts (pan-globin) in cDNAs from both E4 and E6 RBC and nRBC fractions. (C) QPCR data. All genes tested had significantly higher expression in nRBCs by Student's T-test (except for CD51). Hemoglobins are the only genes with significantly higher expression in RBCs. E4 data: left-hand bars, E6 data: right-hand bars.

**Figure 3:**
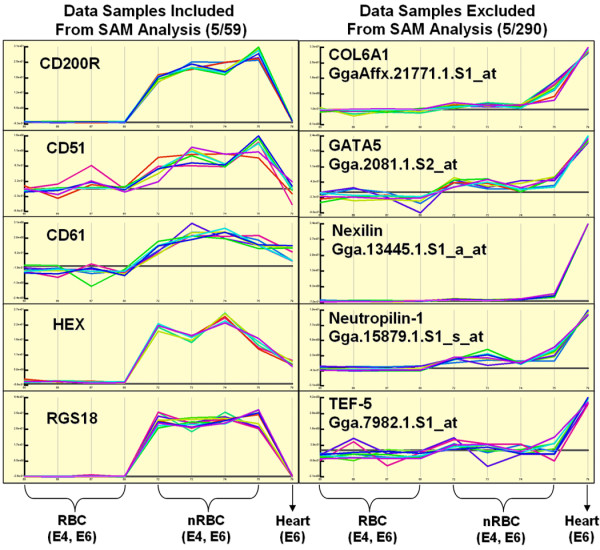
Representative array expression profiles generated by eXintegrator analysis. Example candidate genes kept from TM4 analysis (left) display an observable gradient of low expression in RBC samples and high expression in nRBC samples, and low-to-moderate expression in heart. Example candidate genes discarded from TM4 analysis (right) following examination by eXintegrator display high expression in negative control sample (heart).

Further characterization of these populations by RT-PCR demonstrated that nRBCs had high expression levels of the hematopoietic stem cell antigen CD34, whereas the RBC population lacked expression of this gene (Fig. 2B,C). After these preliminary findings had given validity to our technique, gene expression profiling was performed using Affymetrix Gene Expression Arrays. For RNA isolation, handling time was kept to a minimum and cell collection to lysis for RNA extraction was performed in less than one hour. Consequently, cells were not subjected to long incubation periods on ice, or in serum containing medium, which can alter gene expression, as is the case for other commonly used in techniques such as FACS sorting. RNA from both E4 and E6 RBC and nRBC samples were analyzed by duplicate Affymetrix gene chips, from separate, pooled biological samples (30–100 embryos/array). Comparisons between RBC and nRBC populations were made, and the expression levels of candidate genes were confirmed by PCR (Fig. 2A–C). The resulting array data has been deposited into NCBI Gene Expression Omnibus (GEO) under the accession number GSE9884. We considered genes to be significantly enriched in the nRBC population by the following two criteria: 1) that they are expressed at higher levels in the nRBC than the RBC population by the SAM algorithm; 2) that they are not expressed at high levels in the heart (Fig. 3).

### Hematopoietic Stem Cell (HSC) Associated Genes are Upregulated in nRBCs

Many genes known to be associated with HSCs were found to be preferentially expressed in nRBCs, such as the HSC membrane receptor glycoprotein (GP) CD45 (Tables 1 and 2) [5]. Moreover, transcription factors Ets-1 [6], HEX [7], KLF2 [8] and PU.1 [9], known to be essential for primitive and definitive hematopoiesis, were detected specifically in the nRBC population; as were the signaling molecules BLNK [6], FYN [10], RGS18 [11], Rac2 [12], LYN and SYK [13], VAV3 [14] and the ion channel Slo1 [15]. Additionally, the expression of many integrins, which are known to play an important role in the adhesion and homing of HSCs, were detected in nRBCs. A significant overlapping integrin repertoire was observed between nRBCs and a previous study on adipose derived stromal CD31+ HSCs and includes: CD18 (ITGB2), CD49B (ITGA2), CD49F (ITGA6), CD51 (ITGAV), CD61 (ITGB3/GPIIIa), and the non-integrin cell adhesion molecule CD166 (ALCAM) [16]. In addition to integrins, previous work has established an important role for GPs (e.g. CD34, CD45, CD61) in the adhesion and possibly homing of HSCs, and has demonstrated the expression of the GP receptor CD62L, and the GP Cystatin-7 on HSCs [17,18]. Finally, cell-cell communication required during later differentiation of HSCs in the stroma is known to be mediated by the gap junctional protein connexin 43 (Cx43), another gene detected in the nRBC population [19].

**Table 1:** Genes significantly upregulated in nRBCs. Listed genes are ranked according to expression level in nRBCs shown as Log2 expression values. Genes highlighted in bold were verified by PCR.

**Probe Set ID**	**Gene Name**	**nRBC**	**RBC**
Gga.1039.1.S1_at	**CD61, Integrin Beta 3 (Platelet Glycoprotein IIIa)**	7.53	0.81
Gga.4472.3.S1_x_at	Thymosin Beta 4 TOLL-Like Receptor 7 (TLR7)	7.5	2.35
Gga.9122.1.S1_at	ETS-1 (p54)	7.31	-0.07
Gga.15362.1.S1_at	**RGS18 Regulator of G-protein Signalling 18**	7.11	-3.73
Gga.1187.2.S1_s_at	PINCH-1 hypothetical	7.07	4.02
Gga.5362.1.S1_a_at	**Elfin, PDZ and LIM Domain Protein 1 (PDLIM-1)**	6.81	-0.18
Gga.10042.1.S1_a_at	**CD200 Receptor 1 (CD200R)**	6.78	-1.78
Gga.6387.1.S1_at	p21 Rac2	6.74	-1.68
Gga.2876.1.S2_a_at	VAV3	6.65	-1.71
Gga.19950.1.S1_s_at	Coagulation Factor XIII, A1 Polypeptide	6.49	-2.26
Gga.3316.1.S1_s_at	**HEX probox protein**	6.44	1.91
Gga.12960.1.S1_at	Phospholipase C Gamma 2 (PLCG2)	6.29	-1.81
Gga.4451.1.S1_at	Gelsolin	6.14	0.08
Gga.9413.1.A1_at	Prostaglandin-Endoperoxide Synthase 1 (COX-1)	6.06	2.28
Gga.6665.1.A1_at	Peptide Methionine Sulfoxide Reductase (MSRA)	5.98	-1.16
Gga.514.1.S1_at	Coagulation Factor X Precursor Virus Activating Protease	5.79	-1.51
Gga.7018.1.S1_s_at	Fgd3, FYVE, RhoGEF and PH domain containing 3	5.79	0.51
GgaAffx.13009.1.S1_at	Tumor Necrosis Factor Alpha Induced Protein 8 (TNFAIP-8)	5.52	1.71
Gga.4772.1.S1_at	Connexin 43 (Cx43)	5.46	-3.4
Gga.11854.1.S1_at	Tetraspanin 6 (TSPAN6)	5.44	0.48
Gga.4350.1.S2_at	FYN Oncogene Related to SRC, FGR, YES	5.32	-0.29
Gga.1193.1.S2_at	**CD45, Protein Tyrosine Phosphatase Receptor Type C**	5.24	-1.89
Gga.3828.1.S1_at	ZOV3, Embigin Homolog	5.24	0.61
Gga.16474.1.S1_at	Pleiotrophin (PTN) Osteoblast-Specific Factor 1 (OSF-1)	5.16	-3.79
GgaAffx.21842.1.S1_s_at	Beta Defensin 7 (Gal 7)	5.13	0.18
Gga.11496.1.S1_at	CD62L, L-Selectin	5.09	-1.48
Gga.4225.1.S1_at	Leukocyte Cell-derived Chemotaxin 2 (LECT2)	4.89	0.24
Gga.10034.1.S1_at	Similar to Plasminogen Activator Inhibitor (PAI)	4.85	-3.93
Gga.2734.1.S2_at	CD166, Activated Leukocyte Cell Adhesion Molecule (ALCAM)	4.67	-2.35
Gga.13583.1.S1_at	**CD36, Thrombospondin Receptor (Platelet Glycoprotein IV)**	4.66	-2.74
Gga.2967.1.S2_at	CD49F, VLA6, Integrin Alpha 6	4.47	-0.16
Gga.19342.1.S1_at	Calcium-Activated Potassium Channel Subunit Alpha, Slowpoke (Slo1)	4.45	-3.99
Gga.5758.1.S1_s_at	LYN, Yamaguchi Sarcoma Viral Related Oncogene Homolog (vYES-1)	4.45	-1.42
GgaAffx.12646.1.S1_at	Spleen Tyrosine Kinase (SYK)	4.42	-2.6
Gga.3899.3.S1_a_at	Platelet-Derived Growth Factor Alpha (PDGF-A)	4.38	-2.38
Gga.4832.1.S1_at	Interferon Inducible Transmembrane Protein 3 (IITMP3, Fragilis)	4.25	-0.06
Gga.11657.1.S1_at	Thrombin Receptor, Coagulation Factor II Receptor	4.02	-1.14
Gga.11741.1.S1_a_at	Tissue Factor Pathway Inhibitor (Lipoprotein-Associated Coagulation Inhibitor)	3.98	-2.99
Gga.15893.1.S1_at	**CD49B, Integrin Alpha 2 (Platelet Glycoprotein IIb)**	3.94	-2.95
GgaAffx.22186.2.S1_s_at	Interleukin-1 Receptor Accessory Protein Precursor (IL1RAP)	3.88	-1.26
GgaAffx.13138.1.S1_at	Tumor Necrosis Factor (ligand) Superfamily Member 13b (TNFSF-13b)	3.75	-1.88
GgaAffx.13210.1.S1_at	SRC Like Adaptor (SLAP)	3.62	-4.21
Gga.690.1.S1_at	LY64, MD-1	3.51	-0.47
Gga.3738.1.S1_at	**B-cell Linker (BLNK)**	3.46	-1.18
Gga.4943.1.S1_at	Tumor Necrosis Factor Receptor Superfamily, Member 21	3.32	-1.4
Gga.5743.1.S1_at	LY96, MD-2	3.31	-3.02
GgaAffx.20689.1.S1_at	Spleen Focus Forming Virus (SFFV) Proviral Integration Oncogene (SPI-1, PU.1)	3.29	-2.58
Gga.815.1.S1_at	**CD51, Integrin Alpha V**	3.02	-2.36
GgaAffx.20858.1.S1_at	Cell Adhesion Molecule with Homology to L1CAM Precursor (CHL1)	2.89	-2.24
Gga.2039.1.S1_at	Heme Oxygenase (decycling) 1 (HMOX1)	2.88	-2.69
GgaAffx.11713.1.S1_s_at	Rho Guanine Nucleotide Exchange Factor 3 (GEF-3)	2.74	-1.8
Gga.3070.1.S1_at	CD121A, Interleukin-1 Receptor Type I	2.65	-3.35
Gga.1980.1.S1_s_at	Thrombospondin 4 (THBS4)	2.59	-2.6
Gga.11824.1.S1_at	Cystatin F, Leukocystatin	2.53	-3.57
Gga.4507.1.S1_at	CD18, Integrin Beta 2	2.27	-2.61
GgaAffx.24377.2.S1_s_at	Beta Parvin	2.22	-2.93
Gga.7769.1.S1_at	Fgd5, FYVE, RhoGEF and PH Domain Containing 5	2.15	-2.87
Gga.7528.1.S1_at	Kruppel-like Factor 2 (KLF-2)	2.07	-1.91
GgaAffx.1733.2.S1_s_at	CD62P, Cell Adhesion Molecule LECAM3	1.37	-3.73

**Table 2:** Genes significantly upregulated in nRBCs shown by gene categorization according to functional association. From left to right, genes with a known role in HSCs, myeloid cell lineage, lymphoid cell lineage, other types of non-hematopoietic cells (germ cells, neuronal or cardiac progenitors) or genes with no known role in development. Genes highlighted in bold were verified by PCR.

***HSC (22)***	***Myeloid (17)***	***Lymphoid (10)***	***Germ Line (2)***
**BLNK**	**CD36**	β-Defensin	Fragilis
CD18	**CD49B**	CD121A	Zov3
**CD45**	**Cd51**	HMOX1	
**CD49B**	**CD61**	LECT2	***Neural (2)***
CD49F	CD62P	MD1	PTN
**CD51**	**CD200R**	MD2	TSP4
**CD61**	Coagulation Factor X	TLR7	
CD62L	Coagulation Factor XIII	TNFSF13b	***Cardiac (2)***
CD166	COX-1	TNFSFR21	**Elfin**
**CD200R**	Gelsolin	SLAP	PINCH1
Cx43	PAI-1		
Cystatin F	PDGFA		***Unknown (8)***
ETS-1	PLCG2		β-PARVIN
FYN	PU.1		FGD3
KLF2	Thrombin Receptor		FGD5
LYN	Tissue Factor Pathway Inhibitor		IL1RAP
PU.1	**VAV3**		L1CAM
Rac2			MSRA
**RGS18**			TNFAIP8
Slo1			TSPAN6
SYK			
**VAV3**			

### Myeloid Markers Expressed by nRBCs

In addition to the expression of GPs on HSCs, expression of the platelet GP ligand CD62P (P-selectin), important for HSC adherence [20], and the myeloid GPs CD200R [21] and CD36 [22] were detected in the nRBC population. Other markers of the undifferentiated myeloid lineage including gelsolin [23] and PU.1 were both detected in nRBC fraction. Furthermore, many genes detected in nRBCs can be associated with platelet activation pathways such as, Coagulation Factors X and XIII, COX-1, PAI, PDGF, PLCG2, Tissue Factor Pathway Inhibitor, Thrombin Receptor, and VAV3 [24-27].

### Lymphoid Markers Expressed by nRBCs

Our expression profiling of nRBCs reveals not only the known potential of early circulating embryonic cells towards myeloid and erythroid lineages, but also that of the lymphoid lineage. The expression of leukocyte specific genes which are part of the innate immune system such as the lymphocyte antigens LY64 (MD1) and LY96 (MD2), Toll-like receptor 7 (TLR7) [28] as well as Interleukin 1 Receptor (CD121A) [29], β-Defensin [28], members of the TNF signaling pathway, TNFSF13b (BAFF) and TNFSFR21 (DR6), which can mediate the innate immune response [30], and LECT2, which is involved in neutrophil chemotaxis [31], demonstrate that a lymphoid differentiation potential is already present at the peri-aortic stage, a finding which has been reported previously for similarly staged AGM derived cells in mice [32]. The only gene expressed in nRBCs which has a role in acquired immunity appears to be the Src-like adaptor molecule, SLAP, whose function is to repress surface IgM expression on B-cells [33]. Interestingly, the heme oxygenase-1 (HMOX1) an essential enzyme in heme catabolism, was the only true erythroid associated gene detected in the nRBCs. However, the expression of HMOX1 has been reported in a variety of primitive and definitive white blood cell types as well, although it does not appear to be essential for their development [34].

### Germ Cell, Neural, and Cardiac Markers Expressed by nRBCs

Additional categories of genes that were observed in nRBCs include: Fragilis and Zov3 (associated with germ cells) [35,36], and Pleiotrophin (PTN) and Thrombospondin-4 (THSB4) (found in neuronal stem cells and progenitors) [37,38]. Moreover, despite being negatively screened against genes upregulated in the heart (Fig. 3), Elfin, and PINCH-1, which mark early cardiac cells [39,40], were detected in circulating nRBCs.

### Additional Genes Expressed by nRBCs

Finally, a list of genes with unknown functions has emerged from this screen. Although the Interleukin-1 Receptor Associated Protein (IL1RAP) and TNF-alpha Interacting Protein 8 (TNFAIP8) are likely to be involved in the innate immune response, the other 6 genes remain developmentally uncharacterized, and may provide insight into the function and differentiation of HSCs (Table 2). Interestingly, the Peptide Methionine Sulfoxide Reductase gene (MSRA), which was expressed in nRBCs, may help to protect progenitor cells against oxidative stress [41], although conclusive proof to this end remains to be demonstrated.

### Further Expression Validation: ISH, IHC and FACS

**Figure 4:**
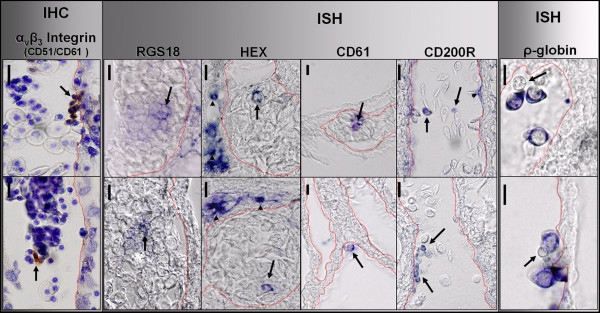
In situ hybridization and histological analyses. From the 12 candidate genes used for QPCR 6 probes were generated that gave some positive signal at earlier embryonic stages; 4 of these stained small numbers of cells in the yolk sac, with the remaining 2 no positive cells were detectable. Embryonic ρ-globin is expressed in the vast majority of circulating cells but is observed to be negative in some rare cells (arrows), whereas CD200R, CD61, HEX, RGS18 and the vitronectin receptor (CD51/61) are expressed in scattered circulating (arrows) and attached cells (arrowheads) throughout the yolk sac vasculature. Red contours outline the vascular lumen. Left and right panels indicate distinct staining of single cells with the indicated probe or antibody. Bars = 20 μm.

**Figure 5:**
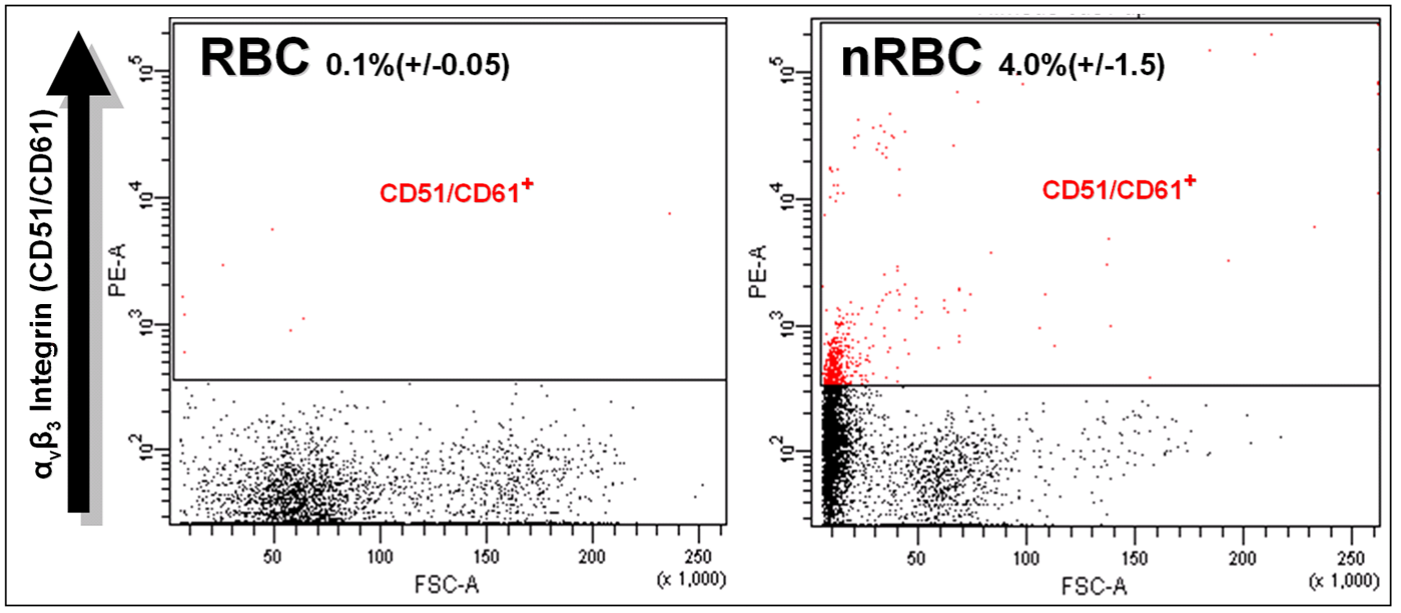
FACS profile of fractionated blood stained with PE-conjugated vitronectin receptor antibody 23C6. The number of positive cells is significantly increased in nRBCs.

Following our expression profiling at the in vitro level, we next verified our expression data for certain interesting candidate nRBC expressed genes, for which QPCR had been performed, by wholemount is situ hybridization (ISH) and immunostaining (IHC) of the yolk sac. At E4 and E6, the time points analyzed, the yolk sac is highly vascularized and provides a receptacle for circulating cells transiting to and from the embryonic and extraembryonic regions. Reproducible labeling of rare positive circulating cells was observed for CD61, CD200R, and HEX, whereas infrequent clusters of positive cells were found to express RGS18 by ISH (Fig. 4). Furthermore, HEX expression was observed in large numbers of mesenchymal cells neighboring blood vessels either containing, or devoid of HEX positive circulating cells. CD200R occasionally labeled cells with an endothelial morphology. IHC for the CD51/CD61 heterodimer or vitronectin receptor (alpha3betaV integrin) revealed expression in rare circulating small rounded cells which were either clumped and associated with the endothelium, or singular. FACS analysis using this antibody demonstrated that 4% of nRBCs are positive for this antigen, whereas RBC staining was negligible (Fig. 5). ρ-globin, which is still prominently expressed in both E4 and E6 RBCs [42], was used as a positive control for yolk sac ISH (Fig. 4). ρ expression was observed in the majority of circulating cells, but was negative in certain infrequent cells presenting non-RBC morphology.

## Conclusion

In summary, gene expression profiling of nRBCs in the chick embryo has revealed the expression of a set of genes indicative of a wide range of hematopoietic stem cells and progenitors primarily of either the erythroid or myeloid lineages present in early circulation. It has indeed been postulated that cells with an "erythromyeloid" potential constitute the first subset of HSCs with potential for liver engraftment and eventual long-term hematopoiesis in the bone marrow [43]. Lastly, the identification of several previously undescribed genes may prompt closer examination of their functions in chick and other model organisms. We, however, do not observe a prominent difference in expression profiles between E4 nRBCs and E6 nRBCs, during which period the second wave of HSC generation is actively taking place in the peri-aortic region, transiting from the initial appearance of intra-aortic clusters at about E4 to the formation of para-aortic foci at E6 [4]. It is therefore unclear whether the nRBCs we detect in E4-6 circulation, with the profiles of hematopoietic cells and progenitors, represent those from yolk sac or peri-aortic cells.

## Methods

### Blood Isolation

Blood was collected from the embryonic ventricles using fine glass microcapillaries. Cells were washed in PBS-EDTA, centrifuged at 1500 g, and separated on a RediGrad:NaCl (9:2.5) density gradient (Amersham) by centrifugation for 20 minutes at 10,000 g. Upper nRBC and lower RBC populations were collected by pipette and placed in RNA lysis buffer or assayed using chemical stains or FACS.

### Benzidine Staining

RBC and nRBC populations were smeared onto glass slides and fixed in 2.5% gluteraldehyde for 1 hr. A 0.1% Benzidine staining solution was then applied (0.001 g/ml benzidine; 0.0068 g/ml imidazole; 0.05 M Tris-HCl, pH7.6; 0.3% H_2_O_2_) for 1 hr at 37°C. Slides were then briefly washed in PBS, dehydrated in ethanol, mounted and photographed.

### RNA Isolation and RT-PCR

Cells were lysed using QIAshredder spin columns and total RNA was extracted using the RNeasy total RNA extraction kit (Qiagen). Equal amounts of total RNA from each sample were converted to first-strand cDNA in parallel with the Superscript III reverse transcriptase synthesis system (Invitrogen). Real-time QPCR was performed using Quantitect SYBR PCR master mix (Qiagen) in a 7900 HT Fast Real-Time PCR System (Applied Biosystems). All PCRs were performed in duplicate with at least 2 biological samples at an annealing temperature of 60°C, using between 30 and 45 amplification cycles. Analysis of the melting curve excluded the amplification of unspecific products. In each QPCR run, a standard curve was generated using duplicate 6-log spanning serial dilutions. PCR products for standard curves were column-purified, measured for DNA concentration, sized by agarose gel electrophoresis, sequenced, aliquoted and stored at -80°C for a maximum of 2 months. Standard curves were calculated by SDS software, and test samples were fitted to the generated curve (Applied Biosystems). Primer sequences are available in Table 3.

**Table 3:** Primer sequences used for PCR and in situ probe generation

**PCR PRIMERS**	
18S rRNA FORWARD	CGAAAGCATTTGCCAAGAAT
18S rRNA REVERSE	GAGTCGGCATCGTTTATGGT
BLNK FORWARD	GAAATCGCCTTCATCCAAAA
BLNK REVERSE	ACCAAGGAGGTATGCTGGTG
CD34 FORWARD	GCAACAACACTGCTCAGCTC
CD34 REVERSE	TTGCTGACACCACCAGATGT
CD36 FORWARD	AAGGAAAGACCCTTGCCAAT
CD36 REVERSE	ATTGCTGCAGTTTCCATTCC
CD45 FORWARD	GCCAAGAGGAGCCATAATCA
CD45 REVERSE	ATCCTGGGTCTCCTGGAATC
CD49B FORWARD	AAAAGAAACGTTGCAAATGAAAT
CD49B REVERSE	GTTTCTGACTTCTCTGCTGCAA
CD51 FORWARD	CATTGAAGGAGACGTGCAAA
CD51 REVERSE	AGTTTGGGTCCAAAGTCGTG
CD61 FORWARD	TTAACAACCCCTTGGCTGTC
CD61 REVERSE	CCACCGAGGTAAGGATGAGA
CD200R FORWARD	TGGTGACTGTCCTTGTGGAA
CD200R REVERSE	GACACAGTGGAGGTGGAACC
Elfin FORWARD	AGCTGCAATAGCCAACCTGT
Elfin REVERSE	GCTCATCTGCACAGCTCTTG
GAPDH FORWARD	TGGGTGTCAACCATGAGAAA
GAPDH REVERSE	CATCCACCGTCTTCTGTGTG
HEX FORWARD	CCCAGATTTCCCATTTCAGA
HEX REVERSE	TACACGAGCAGAGAGGGACA
Pan globin FORWARD	ACCGCCAAGTACCGTTAAGA
Pan globin REVERSE	TTCATCTCATTTGGCTGCTC
RGS18 FORWARD	AAATAAGTGGCAAGCAAAGTTGA
RGS18 REVERSE	CAGCAATAAGTTGCCTGGTTG
Vav3 FORWARD	TCCGCTTGCAAACAATTACA
Vav3 REVERSE	CTCAGGGTGATGGGGAGATA
	
**ISH PROBE PRIMERS**	
CD61 FORWARD	CACCGTGTGTGATGAGAAAA
CD61 REVERSE	ACAGGTTTGATGGTGAAGGA
CD200 FORWARD	TGGCTCTGTACTGCGATGAC
CD200 REVERSE	GAACAGCAAGGGAAAACCAA
CD200 T7 REVERSE	TAATACGACTCACTATAGGGAACAGC
HEX FORWARD	GACTACACGCACGCACTGATC
HEX REVERSE	CAAACTGCTATGTACACGAGCAG
RGS18 FORWARD	CTTCCCACTACCTGCTCTGC
RGS18 REVERSE	GGACCGTGATCGTCTCCTAA
RGS18 T7 REVERSE	TAATACGACTCACTATAGGGGACCGTG

### Affymetrix Array Data Analysis

**Figure 6:**
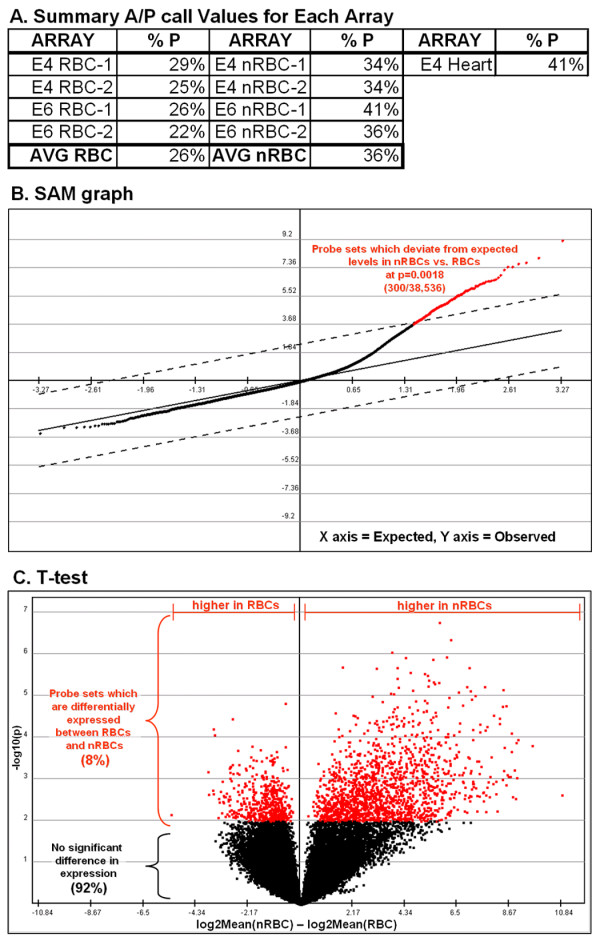
Statistical analyses of the complexity of RBC and nRBC transcriptomes. (A) Summary of present (P) calls for all arrays used in this study as a percentage of all calls (either present, P; absent, A; or missing, M). nRBCs have 10% more P calls than RBCs. Nevertheless, 1/4 of the RBC transcriptome is scored as present. (B) Graphical representation of SAM analysis showing significantly different genes between nRBCs and RBCs in red, which were examined in this study. (C) Grouped T-test between RBCs and nRBCs shows that for a confidence level of 95%, 98% of the hybridization values for probe sets between these two groups are not significantly different.

Amplification of 100 ng of RNA using the Two-Cycle cDNA Synthesis Kit and IVT Labeling Kit (Affymetrix) and subsequent hybridization and scanning was carried out by the Functional Genomics Unit (RIKEN CDB). Pivot raw data files from duplicate microarray experiments were analyzed using the TIGR MultiExperiment Viewer 4.0 (TM4) software package. Briefly, data was log2 transformed, subjected to quantile normalization and analyzed by SAM (Significance Analysis for Microarrays), using 100 random data permutations, and a delta value of 2.5 (p value 0.0018) (Fig. 6). This cutoff was used to minimize false positives, but as a consequence created potential false negatives. A number of previously described hematopoietic markers such as RUNX-1 (Affy ID: Gga.1019.1.S1_at) and cKIT (Affy ID: Gga.606.1.S1_at) were found using a delta value of 0.9 (p value 0.05), but were excluded from our analysis. Hierarchical clustering of significant data points was then carried out by Co-variance algorithm [44]. All significant data points generated by SAM analysis were double checked manually using the eXintegrator software package [45,46] against negative control E6 embryonic heart array data, which was not performed in duplicate. Data points with high expression in heart were eliminated (Fig. 3). Quality control of microarray data, which was well within an acceptable range, was performed using the Bioconductor Affy array analysis suite [47].

### Wholemount ISH and IHC

Yolk sacs from E4 and E6 embryos were collected and fixed in 4% paraformaldehyde, and cut into small pieces to allow greater probe and antibody diffusion. For ISH, samples were processed as described previously [48]. Probes for CD200R and RGS18 were generated by 2 round PCR using primers given in Table 3. Probes for CD61, ρ-globin and HEX were cloned into pGEM-T vector. The probe for embryonic ρ-globin has been described previously [42]. Probe templates were verified by sequencing. RNA probes were generated by either T7 or Sp6 in vitro transcription, and verified by agarose gel electrophoresis. For IHC, all solutions were TBST based. Tissue was blocked for 1 hr (5% sheep serum 1 mg/ml BSA), incubated with mouse anti-human vitronectin antibody, clone 23C6, with known cross reactivity in chicken [49] (BioLegend) at a dilution of 1:40 overnight at 4°C, washed once and blocked for endogenous peroxidase activity, washed again 3×, incubated for 1 hr in secondary HRP conjugated anti-mouse, washed 3×, and developed using DAB solution (Sigma-Aldrich). All tissues were embedded in wax and sectioned to 9 μm on a MICROM HM325 Microtome. IHC samples were counterstained with hematoxylin.

### FACS

Fluorescent Assisted Cell Sorting (FACS) analysis was performed on a BD FACSAria. RBC and nRBCs were separated as described and blocked for 30 min in DMEM/3% FCS (Invitrogen), incubated for 1 hr in either 1:40 PE-conjugated mouse anti-human vitronectin (see IHC) or BD PE-conjugated mouse secondary antibody (negative control, not shown), washed twice in blocking buffer and analyzed. Positive cell counts were observed and recorded using BD FACSDiva software.

## Authors' contributions

BASM and GS designed the experiments; BASM, CA, HT and LMJ performed the experiments; BASM, CA and GS analyzed the data; BASM and GS wrote the paper. All authors have read and approved the final manuscript.
